# How Can I Trust You Again? Repair Behaviours and Trust Recovery After Romantic Betrayal

**DOI:** 10.1111/jmft.70186

**Published:** 2026-11-10

**Authors:** Marco Giacobbi, Fanny Lalot

**Affiliations:** ^1^ Faculty of Psychology University of Basel Basel Basel‐Stadt Switzerland

**Keywords:** attachment, betrayal, couple therapy, infidelity, trust recovery, trust repair

## Abstract

Trust recovery after romantic betrayal is a central concern in couple therapy, yet little is known about which behaviours are most strongly associated with partners' perceived trust repair. We present the results of an online survey of betrayed partners who had attempted reconciliation after romantic infidelity (*N* = 263). Using hierarchical regressions, we tested for the effect of dispositional predictors (attachment anxiety, attachment avoidance, propensity to trust) and betrayed partners' perceptions of five post‐betrayal repair behaviours (transparency, monitoring, remorse, explanation, reconnection) on trust recovery (i.e., differential of trust in the betraying partner now compared to after having discovered the infidelity). Repair behaviours explained substantially more variance in trust recovery than dispositions, which had, at best, rather indirect effects. More specifically, perceived remorse, reconnection, and explanation uniquely predicted trust recovery, whereas transparency and monitoring did not account for additional unique variance once other behaviours were included. These findings suggest trust repair is chiefly associated with what partners *do*, more than with stable predispositions.

Trust serves as a foundational component of intimate relationships, acting as a psychological buffer that facilitates attachment security and allows for vulnerability during distressing life events (Simpson 2007). When betrayal—particularly infidelity—shatters this foundation, couples attempting reconciliation face the challenge of repairing relational trust, that is, rebuilding perceived safety, predictability, and emotional security in the relationship (Fife et al. 2013; Gordon et al. 2004). Yet, although clinical models propose different pathways for repair, empirical research has rarely tested for the simultaneous effect of these behaviours, limiting clarity on which behaviours are uniquely associated with trust recovery when considered together.

The present research focuses on trust restoration, which we operationalise as the change in trust from immediately after discovering the betrayal to the present. Drawing on a recent systematic review of trust repair strategies in romantic relationships, we focus on five theory‐derived behavioural domains commonly discussed in clinical and empirical work: transparency, monitoring, remorse, reconnection, and explanation (Giacobbi and Lalot 2025). To our knowledge, no quantitative study has compared these repair behaviours within the same predictive framework; nor has it tested how dispositional traits are influencing the trust rebuild process, leaving a critical gap in understanding which behaviours, and which dispositions, are most strongly associated with trust recovery after betrayal. Addressing this gap may help determine the relative contribution of theory‐derived repair behaviours and dispositional factors to individual differences in trust recovery.

Building on a cross‐sectional study of people who had recently experienced infidelity (*N* = 263), this paper tests the unique associations of these five repair behaviours with trust recovery, alongside dispositional predictors. Specifically, we examine attachment anxiety, attachment avoidance, and propensity to trust as relatively stable individual differences which are theoretically relevant to interpersonal trust and to how individuals respond to relational threat and trust violations (Campbell and Stanton 2019; Mayer et al. 1995). Examining these predictors alongside perceived repair behaviours also allows us to compare more distal dispositional influences with more proximal, potentially modifiable post‐betrayal relational processes.

## 1 Trust Repair Behaviours

Clinical models of infidelity treatment—including the interpersonal forgiveness model (Fife et al. 2013), attachment injury repair in EFT (Makinen and Johnson 2006), the Gottman Method's Atonement–Attunement–Attachment sequence (Irvine et al. 2024), intersystemic approaches to infidelity treatment (Weeks et al. 2003), and cognitive‐behavioural, trauma‐informed infidelity interventions (Baucom et al. 2006)—offer complementary perspectives on how trust may be repaired after betrayal. Rather than representing competing accounts, we believe these models can be organised around the core relational tasks they emphasise: addressing emotional injury, restoring connection, and rebuilding interpersonal safety.

Here we build on a recent systematic review which endeavoured to consolidate diverse clinical perspectives into specific empirical themes drawn from both quantitative and qualitative research on post‐infidelity couples (Giacobbi and Lalot 2025). This synthesis identified five recurrent behavioural and communicative strategies associated with trust repair across multiple therapeutic traditions, each reflecting one or more relational tasks: (i) transparency, (ii) monitoring, (iii) remorse, (iv) reconnection, and (v) explanation. Conceptually, these five behaviours address different but complementary challenges inherent in trust repair following betrayal, whether facilitated within therapy or enacted spontaneously by couples attempting reconciliation. We briefly describe them in turn below.

*Transparency* refers to increased openness and voluntary sharing of information about daily activities, emotions, and intentions, aimed at reducing ambiguity and restoring a basic sense of predictability. Qualitative accounts describe such openness—about work, contacts, and device use—as evidence of integrity that makes the partner's behaviour predictable again, and identify it as one of the main ways betraying partners re‐establish trustworthiness (Pramudito and Minza 2021).

*Monitoring*, by contrast, involves explicit access to the partner's communications or behaviour (e.g., checking devices or accounts) and reflects efforts to externally regulate uncertainty. While both strategies seek to manage insecurity, transparency relies on relational disclosure, whereas monitoring relies on verification and control. Verification practices are commonly established early in recovery; in one study, some couples described the betraying partner volunteering such information before it was requested and associated this with greater gains in trust, and most reported that these practices became less essential as trust returned (Fife et al. 2023).

*Remorse* captures the betraying partner's acknowledgement of responsibility, expression of regret, and validation of the injured partner's pain. Across therapeutic models, such accountability is understood to address the core relational injury by signalling concern for the partner's suffering and a willingness to repair the harm done. Injured partners describe repeated, authentic expressions of regret and empathy as what gradually eased their sense of ethical imbalance and reduced the urge to retaliate, allowing trust to re‐emerge. Conversely, minimising the betrayal or avoiding discussion of it was experienced as a refusal to take accountability and deepened anger and pain (Walravens and Rober 2024).

*Reconnection* refers to deliberate efforts to re‐establish emotional closeness and positive interaction, such as shared activities, responsiveness, and expressions of care. These efforts aim to rebuild emotional safety and signal renewed relational investment, making closeness feel possible again. In one grounded‐theory study of healed couples, every couple referred to shared time as central to re‐establishing closeness (Fife et al. 2023). Elsewhere, injured partners reported that more frequent communication and expressions of affection led them to appraise the partner and the relationship more positively, strengthening confidence in the partner's fidelity (Pramudito and Minza 2021).

Finally, *explanation* involves providing a coherent and non‐defensive account of why the betrayal occurred. Rather than justifying the behaviour, explanation supports meaning‐making by helping the injured partner make sense of the rupture, restore predictability, and integrate the event into a broader relational narrative. All participants in one narrative study of couples who remained together described it as important to understand why the affair had occurred, and arriving at such an account was among the factors they credited with holding the relationship together (Abrahamson et al. 2012).

Trust repair thus unfolds through specific actions that communicate accountability, emotional safety, and renewed relational investment. Conceptually, the five investigated domains capture different repair tasks that often co‐occur after betrayal. Transparency and monitoring primarily target uncertainty by increasing access to information and reducing ambiguity about daily behaviour (e.g., Abrahamson et al. 2012; Bird et al. 2007); in practice, they tend to function as certainty‐management tools often initiated by the injured partner to mitigate the risk of recurrence (Pramudito and Minza 2021; Walravens and Rober 2024). Remorse targets the relational injury directly by communicating responsibility‐taking and validating the hurt partner's experience, a process that injured partners describe as enabling trust to re‐emerge and confidence to grow over time (Walravens and Rober 2024). Reconnection targets the relational bond by re‐establishing warmth, responsiveness, and shared positive interactions such as shared activities and quality time that make closeness feel safe again (Mitchell et al. 2022; Olson et al. 2002). Finally, explanation targets meaning‐making by helping partners build a coherent account of what happened and why, reducing confusion and restoring predictability through honest disclosure of betrayal causes (Gordon and Baucom 1998; Olson et al. 2002).

### 1.1 Which Repair Behaviours Should Be More Effective?

Giacobbi and Lalot (2025) propose that the five behavioural strategies for trust repair can be organised into two broad categories that reflect fundamentally different mechanisms of action. The first category comprises remorse, reconnection, and explanation (hereafter, connection‐oriented behaviours), which all operate by restoring emotional safety, relational closeness, and shared meaning. The second category comprises transparency and monitoring (hereafter, control‐oriented behaviours), both of which aim to reduce uncertainty and manage insecurity by increasing access to information about the partner's behaviour and whereabouts.

This distinction carries important implications for understanding which behaviours are most likely to support sustained trust recovery, as opposed to merely managing short‐term threat. On the one hand, remorse and reconnection seem to operate as reparative pathways that foster emotional safety and relational healing. When betrayed partners can make sense of why the betrayal occurred, they regain a sense of predictability and coherence essential for rebuilding trust in their partner and the relationship. In line with empirical work identifying reconnection and joint effort as pivotal in recovered couples (Fife et al. 2023), we thus expect connection‐oriented behaviours to be more conducive to trust restoration.

On the other hand, monitoring and the need for transparent behaviour are often driven by attempts to regulate insecurity, and their role—especially that of monitoring—remains uncertain. Empirical and qualitative work describes openness about whereabouts, devices, and contact, including for some couples concrete verification practices such as device or location access and check‐ins, as relevant to rebuilding trust and reassurance, particularly in the early phase of recovery (Abrahamson et al. 2012; Fife et al. 2026; Mitchell et al. 2022; Pramudito and Minza 2021; Walravens and Rober 2024). On the other hand, there is also qualitative evidence that such practices may become less necessary as trust develops (Fife et al. 2023), and other work even indicates that control‐oriented behaviours such as monitoring or pressures for constant accessibility can be perceived as intrusive and may intensify insecurity, thus undermining the repair process in the long run (Reed et al. 2015; Reed et al. 2016). Given this mixed evidence, we do not formulate a directional hypothesis for the effect of transparency and monitoring on trust recovery.

### 1.2 Dispositional and Behavioural Pathways in Trust Repair

Relatively stable individual differences shape both how much trust people extend to a partner and how they respond when that trust is violated (Campbell and Stanton 2019; Mayer et al. 1995; Mikulincer 1998). This makes it important to consider dispositional influences on trust repair alongside behavioural ones. The present study gives primary emphasis to attachment orientations and propensity to trust, because these dispositions bear a direct conceptual relation to trust expectations, sensitivity to relational threat, and willingness to re‐engage in trust after a betrayal.

Within attachment theory, trust is treated as a core component of attachment representations, and the link between insecure attachment and lower dyadic trust is well‐established (Campbell and Stanton 2019). Across five studies using self‐report, diary, and reaction‐time methods, securely attached individuals reported greater trust in their partners and responded to episodes of betrayed trust by talking with the partner, whereas more anxiously attached individuals responded with ruminative worry, Trust and more avoidantly attached individuals by distancing themselves (Mikulincer 1998). Consistent with these findings, attachment anxiety predicts poorer adjustment after a romantic relationship ends, an association largely accounted for by a tendency to dwell on the rupture rather than reflect on it, whereas attachment avoidance showed no comparable association (Saffrey and Ehrenberg 2007). Propensity to trust complements these dimensions by shaping how individuals evaluate interpersonal risk, with those higher in trust propensity more willing to assume others' trustworthiness before having information about them (Mayer et al. 1995; Rotter 1980).

These stable differences may shape how partners experience trust threat and engage with repair efforts. However, because they are distal influences, we argue that observable post‐betrayal behaviours (as more proximal correlates) should be particularly important in predicting trust recovery. In other words, we argue that while dispositional factors may shape how individuals respond to betrayal, observable behaviours are central to determining whether couples successfully rebuild trust.

### 1.3 The Present Study

We rely on cross‐sectional data from individuals who reported being betrayed in a romantic relationship and had attempted to rebuild trust in their partner following the facts. This investigation allows us to test (a) which of the five theory‐derived repair behaviours show the strongest unique associations with trust recovery when considered simultaneously, and (b) how much variance in trust recovery is accounted for by dispositional factors (attachment anxiety, attachment avoidance, propensity to trust) compared with behaviours.

To benchmark the relative contribution of these variables, we estimated three regression models: a dispositions‐only model, a behaviours‐only model, and a hierarchical model in which dispositions were entered first, and behaviours were added in a second step, allowing comparison of explained variance across models and of the incremental variance explained by behaviours beyond dispositions. We aimed to shed light on the following research questions (RQ):

To what extent are perceived transparency, monitoring, remorse, reconnection, and explanation associated with trust recovery at the zero‐order level? When examined simultaneously, which specific repair behaviours demonstrate unique associations with trust recovery?

To what extent are attachment anxiety, attachment avoidance, and propensity to trust (of the betrayed partner) associated with trust recovery at the zero‐order level?

Do perceived repair behaviours explain significant incremental variance in trust recovery above and beyond dispositional factors (when entered in the second step of hierarchical regression)?

## 2 Methods

This study adhered to the STROBE guidelines for reporting observational research (Von Elm et al. 2007), the APA Journal Article Reporting Standards for Quantitative Research (JARS‐Quant; Appelbaum et al. 2018), and the Transparency and Openness Promotion (TOP) standards (Nosek et al. 2015). The study was not preregistered due to its exploratory nature. Anonymised data, code for analysis, and supplementary materials are publicly available on the OSF: https://osf.io/t4cwy.

### 2.1 Participants and Procedure

The study was introduced as an investigation of “the strategies that may or may not help restoring trust in a couple following an episode of betrayal.” An online investigation hosted on Qualtrics it was advertised on LinkedIn, Facebook, Instagram, TikTok, and Reddit. It was available in English and Italian; the Italian version was produced via professional translation and independent back‐translation of the English version. Data were collected between 23 July and 29 October, 2025. Recruitment targeted adults who had experienced romantic betrayal and attempted reconciliation (see exclusion criteria below). Participation was voluntary; however, as compensation for their time, participants received a digital informational e‐book upon completing the study. This provided them with evidence‐based guidance on navigating relational betrayal, focusing on the five restorative domains identified in recent research: transparency, monitoring, remorse, reconnection, and explanation. The study protocol was approved by the Ethics Committee of the Faculty of Psychology at the University of Basel. All participants provided informed consent electronically. Informed by simulation studies that show that correlations as small as 0.10 stabilise at a sample size of 250 (with a width of the corridor of stability *w* = 0.10, power = 0.80; see Schönbrodt and Perugini 2013), we aimed to recruit a minimum of 250 participants.

A total of 757 individuals accessed the survey, although many stopped early and did not finish it. We first excluded 293 respondents who completed less than 95% of the survey, leaving 464 participants. We then excluded 41 respondents who failed one or both attention checks embedded in the questionnaire, leaving 423. Next, we excluded 103 respondents who did not identify as the betrayed partner (and were therefore not eligible for the betrayed‐partner analyses), leaving 320. We then excluded 53 respondents who indicated (via an honesty/eligibility check) that they had not attempted to rebuild trust in their relationship, leaving 267. Finally, we excluded 4 respondents who reported completing the survey not seriously on the seriousness check (1–7 scale; score < 6), yielding a final sample size for analysis of *N* = 263.

They were 66 men, 196 women, and 1 non‐binary with a mean age of 48.83 years (SD = 11.05; range = 19–81 years). Participants had been in the relationship for an average duration of 15.51 years (SD = 11.54, *Mdn* = 15.00) when the betrayal occurred, and the betrayal had occurred an average of 37.70 months before survey completion (SD = 56.80, *Mdn* = 22.00). Overall, 68.8% reported having at least one child with the partner; 68.4% were still together at the time of the survey; and 46.4% reported having attended couple therapy since the betrayal. The survey was completed in Italian by 79.3% of participants and in English by 20.7%. Formal educational attainment and legal marital status were not assessed.

### 2.2 Measures

#### 2.2.1 Trust and Trust Recovery Index

Participants reported how much they trusted their partner at three time points in their history, using single‐item measures (1 = not at all to 5 = completely): before betrayal (“How much did you trust your partner before the betrayal?”), after betrayal (“Right after finding out about the betrayal, how much did you trust your partner?”), and presently (“How much do you trust your partner now?”). The primary outcome variable, trust recovery, was calculated as Trust Now minus Trust After Betrayal, representing the degree of trust recovery from the post‐discovery level. This operationalisation is consistent with infidelity‐recovery research conceptualising recovery as a process of rebuilding trust following disclosure (Olson et al. 2002), as well as treatment research assessing trust at pre‐ and post‐treatment (Irvine et al. 2024). The retrospective difference score was therefore intended to capture perceived trust regained from the immediate post‐discovery level rather than only current absolute trust.

Although no study to our knowledge has validated this exact retrospective trust‐recovery difference score, research within the romantic‐relationship domain indicates that single‐item global measures can show satisfactory psychometric performance for some relationship constructs, including satisfaction, love, and commitment (Niehuis et al. 2024; Fülöp et al. 2022).

#### 2.2.2 Attachment

The Experiences in Close Relationships Scale‐Short Form (ECR‐S; Wei et al. 2007) measured adult attachment using 12 items. The ECR‐S covers two dimensions: Attachment Anxiety (e.g., “I often worry that my partner may leave me”) and Attachment Avoidance (e.g., “I feel uncomfortable depending on a partner”) with 6 items per dimension. Items were rated on a 7‐point Likert scale (1 = strongly disagree, 7 = strongly agree). In the present sample, Cronbach's α was 0.75 for Attachment Anxiety and 0.67 for Attachment Avoidance.

#### 2.2.3 Propensity to Trust

The Propensity to Trust Scale (Frazier et al. 2013) assessed one's general tendency to trust others (e.g., “My tendency to trust others is high”). This measure captures dispositional trust as a stable individual difference. The four‐item scale was rated on a 5‐point scale (1 = strongly disagree to 5 = strongly agree).^1^ In the present sample, internal consistency was good (*α* = 0.88).

#### 2.2.4 Perceived Trust‐Restoring Behaviours

Building on the systematic review by Giacobbi and Lalot (2025), we developed 15 items to assess the betrayed person's perceptions of five specific trust restoration strategies or behaviour as enacted by their partner (3 items by behaviour): *Transparency and Honesty* (e.g., “My partner has been completely open about their activities and whereabouts”), *Monitoring* (e.g., “I could check my partner's phone, email, or social media accounts”), *Remorse and Accountability* (e.g., “My partner has taken full responsibility for the betrayal without making excuses”), *Reconnection* (e.g., “We have made efforts to spend quality time together doing activities we both enjoy”), *Explanation and Reasons* (e.g., “My partner has explained what led them to betray my trust”).

All items were rated on a 5‐point scale (1 = *never* to 5 = *all the time*), with a “doesn't apply” response option to indicate that a behaviour was not relevant to the participant's situation (e.g., not feasible, not requested, or not applicable in their relationship context). “Doesn't apply” responses were coded as missing values rather than as low behavioural endorsement, to avoid conflating non‐applicability with low levels of the behaviour. Composite scores were computed as the mean of available items, requiring at least two of the three items per behavioural domain to be non‐missing; otherwise, the composite was set to missing. This occurred for 7 participants on the transparency/honesty composite, 21 on monitoring, 2 on remorse/accountability, 1 on reconnection, and 3 on explanation. Descriptive statistics and reliability indices for all variables are reported in Table 1. In the present sample, internal consistency was good across the five behavioural domains: Transparency (*α* = 0.80), Monitoring (*α* = 0.92), Remorse/Accountability (*α* = 0.84), Reconnection (*α* = 0.92), and Explanation/Reasons (α = 0.87).

**Table 1:** Descriptive statistics and reliability for study variables.

Measure	*k* (items)	Response scale	Valid *N*	*M*	SD	*α*
Trust before betrayal	1	1–5	263	4.57	0.83	—
Trust after betrayal	1	1–5	263	1.44	0.79	—
Trust now	1	1–5	263	2.40	1.21	—
Trust Recovery	—	−4 – +4	263	0.96	1.26	—
Transparency/Honesty	3	1–5	256	2.98	1.27	0.80
Monitoring	3	1–5	233	3.03	1.50	0.92
Remorse/Accountability	3	1–5	261	3.18	1.24	0.84
Reconnection	3	1–5	262	3.30	1.27	0.92
Explanation/Reasons	3	1–5	260	3.00	1.25	0.87
Attachment anxiety	6	1–7	263	4.27	1.11	0.75
Attachment avoidance	6	1–7	263	2.75	0.88	0.67
Propensity to trust	4	1–5	263	3.71	0.94	0.88

*Note:* Trust recovery = Trust now − Trust after betrayal. Valid N varies due to “doesn't apply” responses coded as missing and the ≥ 2‐of‐3 item rule for composites. *α* = Cronbach's alpha.

To assess dimensionality, we conducted confirmatory factor analyses for each multi‐item measure. The five‐factor model of the perceived repair behaviours and the one‐factor model of propensity to trust showed acceptable fit (CFI ≥ 0.95, TLI ≥ 0.94, RMSEA ≤ 0.09). Detailed measurement‐model results are available on the OSF.

### 2.3 Data Analysis

All analyses were conducted in JASP (Version 0.95.3; JASP Team 2025). The primary outcome was trust recovery (Trust Recovery = Trust Now − Trust After Betrayal). Because “doesn't apply” responses for behavioural items were coded as missing, and behavioural composites were computed only when sufficient item data were available (see above), the effective sample size varied by model. For regression analyses, missing data on predictors were handled using listwise deletion, yielding *n* = 229 for the main comparative models reported in the Results section.

To evaluate the relative contribution of dispositional versus behavioural predictors, we estimated three hierarchical multiple regression models (two‐tailed, *α* = 0.05): (1) a dispositions‐only model including attachment anxiety, attachment avoidance, and propensity to trust; (2) a behaviours‐only model including the five repair behaviours (transparency, monitoring, remorse, reconnection, explanation); and (3) a dispositions‐and‐behaviours model. Model comparisons focused on *R* ^2^ and adjusted *R* ^2^, and on the incremental variance explained by behaviours over dispositions (Δ*R* ^2^ and *F*‐change). Regression coefficients are reported with unstandardised estimates (*b*), standard errors (*SE*), standardised coefficients (*β*), and *p*‐values. Prior to modelling, we screened variables for implausible values and inspected regression assumptions using residual diagnostics. Multicollinearity was evaluated via intercorrelations among predictors and variance inflation indicators; no violations were judged severe enough to preclude interpretation of regression estimates. Model‐specific *N*_s_ and full outputs are reported in the Results.

## 3 Results

Trust ratings showed the expected pattern: high trust before betrayal, dramatic decline immediately after discovery, and partial recovery at the time of survey completion. The mean trust recovery score indicated that, on age, participants recovered approximately one scale point from their post‐betrayal lower level, though substantial individual variation existed.

### 3.1 Bivariate Correlations

Table 2 reports zero‐order correlations among trust recovery, the five repair behaviours, and dispositional predictors. Trust recovery was positively correlated with all five behaviours, with the largest associations observed for remorse/accountability and reconnection. Transparency and explanation showed medium‐to‐large correlations, whereas monitoring showed the smallest (though still statistically significant) association.

**Table 2:** Zero‐order correlations among trust recovery, repair behaviours, and dispositional predictors.

Variable	1	2	3	4	5	6	7	8
1. Trust Recovery	—							
2. Transparency/Honesty	0.44***	—						
3. Monitoring	0.39***	0.81***	—					
4. Remorse/Accountability	0.55***	0.63***	0.62***	—				
5. Reconnection	0.52***	0.58***	0.56***	0.70***	—			
6. Explanation/Reasons	0.45***	0.44***	0.44***	0.60***	0.54***	—		
7. Attachment anxiety	−0.16*	−0.24***	−0.26***	−0.26***	−0.22***	−0.31***	—	
8. Attachment avoidance	−0.12	−0.14*	−0.15*	−0.19**	−0.07	−0.22***	0.12*	—
9. Propensity to trust	−0.10	−0.10	−0.08	−0.12*	−0.05	−0.14*	0.02	0.03

*Note:* Values are Pearson's r. Trust recovery = trust now − trust after betrayal.

* *p* < 0.05

** *p* < 0.01

*** *p* < 0.001.

Dispositional variables showed modest associations with trust recovery and with the behavioural strategies. Attachment anxiety correlated negatively with all five behavioural domains and with trust recovery, and attachment avoidance showed smaller negative associations with several behaviours. Propensity to trust also showed a small negative association with explanation and remorse/accountability.

### 3.2 Multiple Regression Models Predicting Trust Recovery

To evaluate the relative contribution of dispositional versus behavioural predictors, we estimated three hierarchical regression models, using listwise deletion across predictors (model *N* = 229). Table 3 summarises model fit (*R* ^2^, adjusted *R* ^2^) for the dispositions‐only, behaviours‐only, and hierarchical models, and reports the incremental variance explained when behaviours were added after dispositions (Δ*R* ^2^ and *F*‐change).

**Table 3:** Hierarchical regression models predicting trust recovery.

	Model 1 (dispositions only)					Model 2 (behaviours only)					Model 3 (dispositions + behaviours)				
	*b*	*SE*	*β*	*t*	*p*	*b*	*SE*	*β*	*t*	*p*	*b*	*SE*	*β*	*t*	*p*
Propensity to trust	−0.156	0.087	−0.118	−1.797	0.074	—	—	—	—	—	−0.054	0.071	−0.040	−0.757	0.450
Attachment anxiety	−0.023	0.013	−0.122	−1.847	0.066	—	—	—	—	—	0.006	0.011	0.031	0.559	0.577
Attachment avoidance	−0.022	0.016	−0.092	−1.403	0.162	—	—	—	—	—	0.004	0.013	0.016	0.283	0.777
Transparency/Honesty	—	—	—	—	—	0.149	0.094	0.149	1.595	0.112	0.150	0.094	0.149	1.589	0.113
Monitoring	—	—	—	—	—	−0.079	0.078	−0.093	−1.011	0.313	−0.076	0.079	−0.089	−0.970	0.333
Remorse/Accountability	—	—	—	—	—	0.260	0.088	0.251	2.956	0.003	0.261	0.089	0.252	2.926	0.004
Reconnection	—	—	—	—	—	0.250	0.078	0.248	3.210	0.002	0.249	0.080	0.248	3.130	0.002
Explanation/Reasons	—	—	—	—	—	0.177	0.069	0.172	2.548	0.011	0.181	0.072	0.176	2.500	0.013
*R* ^2^	0.041					0.395					0.397				
Adjusted *R* ^2^	0.029					0.381					0.375				
Δ*R* ^2^	—					—					0.356				
F‐change (df1, df2), p	—					—					26.01 (5, 220), < 0.001				

*Note:* N = 229 (listwise deletion across predictors). Trust recovery = Trust now − Trust after betrayal. b = unstandardised coefficient; SE = standard error; *β* = standardised coefficient

The dispositions‐only model (attachment anxiety, attachment avoidance, propensity to trust) explained a small proportion of variance in trust recovery (*R* ^2^ = 0.041; adj. *R* ^2^ = 0.029). The behaviours‐only model explained substantially more variance (*R* ^2^ = 0.395; adj. *R* ^2^ = 0.381). Adding behaviours after dispositions produced a large increase in explained variance (Δ*R* ^2^ = 0.356), *F*‐change (5, 220) = 26.01, *p* < 0.001, yielding a final model of *R* ^2^ = 0.397 (adj. *R* ^2^ = 0.375).

In the full model (dispositions + behaviours), remorse/accountability, reconnection, and explanation emerged as unique predictors of trust recovery, whereas transparency and monitoring did not contribute uniquely once the other behavioural domains were included. This suggests that the zero‐order associations of transparency and monitoring with trust recovery were largely accounted for by their shared variance with remorse, reconnection, and explanation.

In an exploratory manner, we also tested whether dispositional characteristics would relate to the perception of specific repair behaviours, which would in turn relate to trust (i.e., a form of mediation analysis going from dispositions through behaviours to trust repair; the full analysis is reported in Supplementary Material, SM1). It should be noted from the outset, however, that given the cross‐sectional nature of the study, this analysis cannot be interpreted as evidence for causal links; it merely shows which variables share common variance in explaining trust recovery. A Structural Equation Model including all three dispositional predictors, five behaviours, and the trust recovery index showed that attachment anxiety was negatively associated with all five perceived repair‐behaviour domains. In addition, attachment avoidance and trust propensity were both associated with lower remorse and explanation. In this model, attachment anxiety and trust propensity were both associated with lower trust recovery; attachment avoidance, however, was not. Tests of indirect effects suggested that trust propensity might have lowered trust recovery through decreased explanation (but not through the other behaviours). Furthermore, attachment anxiety might have lowered trust recovery through a mix of decreased transparency, remorse, reconnection, and explanation. These exploratory associations are consistent with the possibility that dispositions may shape more proximal post‐betrayal relational processes, although we must stress again that the cross‐sectional design cannot establish temporal or causal relations.

## 4 Discussion

This study examined how five theory‐derived trust‐restoring behaviours (transparency, monitoring, remorse, reconnection, and explanation) and three dispositional tendencies (attachment anxiety, attachment avoidance, and propensity to trust) would relate to self‐reported trust recovery in romantic relationships following betrayal. In an online survey of betrayed partners who had attempted reconciliation after infidelity, we operationalised trust recovery as the change in trust from immediately after discovering the betrayal to the time of the survey.

Across the three regression models, perceived repair behaviours (as enacted by the betraying partner) accounted for substantially more variance in trust recovery than dispositional factors of the betrayed partner. Furthermore, only a subset of behaviours showed unique associations when considered simultaneously. Specifically, remorse/accountability, reconnection, and explanation emerged as the strongest unique correlates of trust recovery, whereas transparency and monitoring did not contribute uniquely once the other behaviours were included (Table 3). Dispositional factors, on the other hand, only showed modest associations with trust recovery and with (some) repair behaviours (Table 2).

### 4.1 Connection‐Oriented Behaviours and Trust Recovery

The most important finding of the present study is that connection‐oriented behaviours (i.e., remorse and accountability, reconnection through shared activities and emotional attunement, and explanation of reasons) showed substantially stronger unique associations with trust recovery than control‐oriented behaviours (i.e., transparency and monitoring). That remorse and accountability emerge as the strongest predictor of trust recovery underscores the value of making remorse visible through consistent, observable accountability and responsiveness over time, rather than relying on reassurance alone. This aligns with attachment‐based accounts of partners serving as a safe haven and secure base for each other (Johnson 2004), grounded in Bowlby's description of the attachment figure as a secure base from which one can explore and to whom one can return when upset or frightened (Bowlby 1988). When betrayers demonstrate genuine remorse, take accountability without defensiveness, and validate the betrayed partner's pain, they may address the impasse created by the attachment injury, a situation where the attachment figure is perceived as both the source of and the solution to emotional pain (Johnson 2004). This pattern is consistent with qualitative work which identified accountability as an important component of recovery after infidelity (Mitchell et al. 2022; Walravens and Rober 2024).

Reconnection and emotional attunement also emerged as an equally powerful predictor of trust recovery. One parsimonious reading is that trust is rebuilt by repeated experiences of emotional accessibility—moments in which closeness feels safe again rather than demanded. In infidelity‐focused work, fostering empathy and reducing emotional distance is often treated as central to helping couples begin reconnecting and bridge the chasm created by the affair (Fife et al. 2013). Qualitative evidence has also identified intentional reconnection and emotional communication as part of successful post‐infidelity healing (Fife et al. 2023; Mitchell et al. 2022). At the same time, reconnection can derail when it functions as pressure to “move on”: if shared activities become a substitute for responsiveness or accountability, they may be experienced as avoidance rather than repair.

Explanation and reasons also remained uniquely associated with trust recovery, consistent with the idea that recovery is not only behavioural but also interpretive: partners often need a coherent account that restores predictability after a rupture dominated by secrecy and ambiguity. Reibstein (2013) highlights that secrecy is central to the harm of affairs because it shatters the relationship's attachment hierarchy, positioning disclosure and meaning‐making—effectively the construction of an “explanation”—as the essential tasks for restoring safety. In addition, Gordon and Baucom's synthesised model frames betrayal recovery as moving from the immediate impact phase into a “search for meaning” phase, where putting the betrayal into context reduces confusion and supports forward movement (Gordon and Baucom 1998; see also Abrahamson et al. 2012; Olson et al. 2002). Importantly, this does not imply that “more detail” is always better: treatment accounts emphasise pacing disclosure and containing repetitive questioning when it becomes obsessive fact‐finding that compounds distress rather than supporting healing (Fife et al. 2013).

### 4.2 Transparency and Monitoring: Shared Rather Than Unique Associations

Both transparency and monitoring showed positive zero‐order associations with trust recovery, consistent with the wider infidelity literature describing openness, verification, and reassurance as potentially important during recovery (Abrahamson et al. 2012; Fife et al. 2026; Mitchell et al. 2022; Pramudito and Minza 2021; Walravens and Rober 2024). Once the other repair behaviours were taken into account, however, neither accounted for unique variance. One reason they may add little once other repair behaviours are in place is that verification cannot supply the reassurance for which it is sought: because evidence of continued contact can easily be concealed, not finding evidence does not establish that there is nothing to find. Indeed, clinical accounts describe betrayed partners who go on checking without ever reaching the confirmation they are looking for (Hertlein et al. 2017).

Rather than building trust, monitoring may thus function as a substitute for it: injured partners describe checking as compensating for trust that is not yet present, and report gradually relinquishing the need to know their partner's whereabouts as trust returns (Walravens and Rober 2024). Where checking continues, however, the confidence partners report may remain conditional upon it (Pramudito and Minza 2021), and injured partners who choose to stay describe rebuilding trust as something they must actively negotiate rather than verify (Mitchell et al. 2026). Outside the infidelity context, electronic monitoring in romantic relationships has been linked to attachment anxiety and to behaviour experienced as intrusive (Reed et al. 2015; Reed et al. 2016). These practices may therefore be most helpful in the early stages of the recovery process, becoming less essential or even damaging as trust develops again (Fife et al. 2023).

At present, our cross‐sectional data cannot determine with certainty whether transparency or monitoring is helpful or harmful at any particular stage of the relationship. They suggest only that the role of these behaviours, whichever it may be, is not as central to trust recovery as that of the connection‐oriented behaviours.

### 4.3 Attachment Style and Propensity to Trust: No Strong Evidence for the Role of Dispositional Factors

Our findings only identified weak relations between trust and attachment anxiety, avoidance, and propensity to trust, and what zero‐order correlation emerged did not survive the multiple regression test. At first glance, one may conclude that attachment processes are unimportant to the trust repair process. However, we believe it is too early for such an assessment. Indeed, the ECR‐S as used in our study assesses general attachment anxiety and avoidance, but does not directly assess whether the respondent's particular partner is currently experienced as a “safe haven” or secure base. Betrayal may disrupt partner‐specific security rather than one's general attachment style, something that future studies would need to assess before we can conclude with some certainty about the role, or absence thereof, of attachment styles (Bowlby 1988; Johnson 2004; Makinen and Johnson 2006). This distinction is not merely conceptual: partner‐specific attachment measures have been found to predict adjustment more strongly than general ones (Saffrey and Ehrenberg 2007), and current work treats people as holding one global attachment representation alongside relationship‐specific representations nested within it (Campbell and Stanton 2019).

A similar consideration applies to propensity to trust, which was assessed as a general disposition to trust others rather than as trust extended to this particular partner. Because our outcome concerns trust in one specific person following a specific violation by that person, a general tendency to trust unfamiliar others may simply be too distal to register. As for attachment tendencies, it would be helpful for future research to consider one's tendency to trust people from different distinct categories, thus separating one's partner from, for example, one's family and friends (Couch and Jones 1997).

In spite of these methodological considerations, it is also worth noting that the exploratory structural equation model revealed indirect effects of attachment anxiety on trust recovery through decreased transparency, remorse, reconnection, and explanation, as well as an indirect effect of trust propensity through decreased explanation (see SM1). While these exploratory findings cannot be considered evidence for causal relationships given the cross‐sectional nature of the data, they suggest that the influence of dispositional predictors might be indirect rather than direct, something that future studies may need to investigate further.

### 4.4 Limitations and Directions for Future Research

Several limitations of the present research should be noted. First, online self‐selection likely attracted individuals already motivated to reflect on betrayal, reducing sample representativeness. Retrospective trust ratings may be affected by recall bias, and exclusive reliance on self‐report could have introduced common‐method and social‐desirability biases. Future observational studies coding couple interactions for remorse expression, reconnection attempts, and explanation quality would allow testing whether behaviours predict trust recovery independently of subjective perception. In addition, as noted above, the cross‐sectional design prevents causal inference and does not establish temporal ordering among dispositions, repair behaviours, and trust recovery. Repair behaviours may contribute to trust recovery, but higher current trust could also shape how participants recall and evaluate past behaviours. Longitudinal designs following couples from discovery through recovery—with repeated assessments of behaviour and trust—would allow for stronger tests of directionality and change over time. In addition, experimental or quasi‐experimental designs testing targeted therapeutic interventions could more directly test the relative contribution of connection‐oriented and control‐oriented behaviours examined here.

Further, the sample was predominantly female (74.5%) and predominantly Italian‐language (79.3%), likely reflecting a largely Italian cultural context, which may limit generalisability. Cultural norms may influence not only decisions about reconciliation after infidelity, but also the meaning attributed to specific repair behaviours, including disclosure, monitoring, expressions of remorse, relational privacy, and expectations about remaining in the relationship. The extent to which these cultural factors shaped the present findings cannot be determined, and cross‐cultural replication is therefore needed.

Finally, the use of single‐item trust ratings limits psychometric precision. Previous research found that single‐item measures can show good psychometric performance for romantic‐relationship constructs (Niehuis et al. 2024; Fülöp et al. 2022), but this likely varies by construct. The present retrospective trust‐recovery difference score had not itself been psychometrically validated; further, it cannot distinguish among different facets of relational trust. Future studies should employ validated multi‐item trust scales and repeated prospective assessment to provide a more precise test of trust change over time.

### 4.5 Implications for Therapeutic Practice

Clinically, the present pattern of results supports prioritising interventions that increase specific repair behaviours—while using dispositional profiles mainly to anticipate barriers, pacing needs, and common derailers (e.g., hypervigilance or emotional distancing). Therapists may therefore encourage, but also supervise and time‐limit, transparency and monitoring agreements during crisis stabilisation, Indeed, prolonged surveillance sustains an asymmetry between partners that treatment should be working to dissolve (Baucom et al. 2006; Hertlein et al. 2017). Therapists must then shift focus to facilitating genuine remorse, emotional reconnection, and coherent meaning‐making. Effective therapy should work toward a graduated, collaboratively decided reduction of external verification as internal trust rebuilds, with the pace set by the betrayed partner rather than imposed.

With this in mind, we believe it is important to stress that couples should be helped in understanding that while monitoring may provide temporary reassurance, lasting trust recovery requires additional and deeper emotional work, one that relies on accountability, reconnection, and meaning‐making. Interventions should focus on facilitating genuine remorse expression by the betrayer and validating the betrayed partner's pain. This is the relational injury that EFT frames as an attachment injury requiring repair before the bond can be re‐established (Makinen and Johnson 2006). Such work may be facilitated through structured accountability techniques, such as those outlined in the Gottman Method's atonement phase (Gottman and Gottman 2008; Irvine et al. 2024) and cognitive‐behavioural infidelity protocols (Baucom et al. 2006), including guided apology exercises, explicit responsibility‐taking dialogues, and collaborative processing of the betrayed partner's most pressing questions.

Second, our findings underscore the centrality of reconnection in trust restoration. Clinicians can assign structured shared activities—not as avoidance of the betrayal, but as deliberate opportunities to re‐establish positive interaction patterns and signal renewed commitment. Qualitative work with couples who report successful recovery after infidelity identifies intentional reconnection practices and deep emotional communication as core mechanisms of healing (Fife et al. 2023). In practice, reconnection work should be intentional rather than incidental. Therapists can draw on EFT's in‐session enactments to build new bonding cycles through vulnerability and responsive engagement (Johnson 2019), and assign the structured between‐session rituals central to the Gottman Method's attachment phase (Irvine et al. 2024)—both designed to create new positive relational experiences that counterbalance the injury of betrayal.

Third, explanation emerged as a significant correlate of trust recovery, highlighting that betrayed partners often need a coherent understanding of why the betrayal occurred. This corresponds to the “search for meaning” phase in Gordon and Baucom's (1998) model, in which putting the betrayal into context reduces confusion and allows the couple to move forward. Nonetheless, this process must be paced and clinically contained. Therapists should help betrayers provide honest, non‐defensive accounts that acknowledge responsibility while offering context, and support betrayed partners in hearing these explanations without becoming overwhelmed by renewed pain. Importantly, explanation should not drift into justification: infidelity treatment frameworks are explicit that the act remains unjustifiable and that efforts to repair must avoid excuses, minimisation, or defensive reframing (Fife et al. 2008). Balancing transparency with emotional safety requires skilled clinical judgement.

Finally, given that attachment insecurity is linked to hyperactivation (anxiety) and deactivation (avoidance) responses, clinicians should assess each partner's attachment orientation early and adjust pacing and structure accordingly. Anxiously attached partners may need specific support to regulate arousal, while avoidantly attached partners may require help tolerating vulnerability during these discussions. Using such profiles to plan scaffolding and session structure may improve engagement in the behavioural tasks most closely linked with trust recovery.

## Author Contributions

**Marco Giacobbi:** conceptualisation, methodology, investigation, data curation, formal analysis, writing – original draft, writing – review and editing. **Fanny Lalot:** conceptualisation, methodology, formal analysis, supervision, writing – review and editing.

## Policy on Using ChatGPT and Similar AI Tools

The authors used a large language model‐based tool to assist with English language editing. All substantive content, analyses, and interpretations are the authors' own responsibility.

## Ethics Statement

The study protocol was approved by the Ethics Committee of the Faculty of Psychology at the University of Basel (application number: 009‐25‐2). All participants provided informed consent electronically.

## Supporting information


Supporting File

## Data Availability

Anonymised data, code for analysis, and supplementary materialsare openly available in the OSF at https://osf.io/t4cwy/.

## Footnotes

1 For transparency purposes, we wish to disclose that the questionnaire also included a measure of personality. Specifically, we included the Big Five Inventory‐2–Extra Short Form (BFI‐2‐XS; Soto and John 2017), which assesses five personality domains using 15 items (3 per domain). However, in the present sample, internal consistencies were low to modest for several domains (Extraversion *α* = 0.47; Agreeableness *α* = 0.45; Conscientiousness *α* = 0.62; Negative Emotionality *α* = 0.64; Open‐Mindedness *α* = 0.36), indicating limited shared variance among items and raising concerns about whether domain aggregates meaningfully captured the intended constructs. Given these reliability concerns, BFI‐2‐XS traits were excluded from the primary regression models, and dispositional predictors were restricted to attachment anxiety, attachment avoidance, and propensity to trust.
