# Supplementary material for: How Can I Trust You Again? Repair Behaviours and Trust Recovery After Romantic Betrayal
Source: J Marital Fam Ther. 2026 Nov 10;53(1):e70186. doi: 10.1111/jmft.70186 (PMC13749597; doi:10.1111/jmft.70186)
Supplement: Supplementary file 1 [file JMFT-53-0-s001.docx]

**Repair Behaviours and Trust Recovery After Romantic Betrayal**

**Electronic Supplementary Materials**

# **SM1. Exploratory Mediation Analysis**

In an exploratory manner, we tested whether dispositional characteristics would relate to the perception of specific repair behaviours, which would in turn relate to trust (i.e. a form of mediation analysis going from dispositions through behaviours to trust repair). It should be noted from the outset that, given the cross-sectional nature of the study, this analysis cannot be interpreted as evidence for causal links; it merely shows which variables share common variance in explaining trust recovery.

**Analysis Strategy**

We relied on Structural Equation Modelling (SEM) and tested a model where the dispositional predictors (attachment avoidance, attachment anxiety, and trust propensity) were related to trust through the proximal predictors (i.e., the five repairing behaviours). All indicators were allowed to covary. An initial model including both the measurement model and the regression model failed to converge. We thus relied on aggregated indicators of all constructs and tested the regression model only. We ran a joint-significance test to examine the component paths, then relied on a bootstrap resampling method to examine the magnitude of the indirect effect (percentile bootstrap confidence intervals; Yzerbyt et al., 2018). Analyses were conducted in RStudio 2026.07.1+147 with package *lavaan* (Rosseel, 2012).

**Results**

Since indicators are allowed to covary in a SEM, regression results can slightly differ from those obtained in a LS multiple regression analysis. Here, the results across the two methods were mostly aligned: trust recovery was positively related to remorse (β = .28***), reconnection (β = .28***), and explanation (β = .20**) – see Table S1 below. In the SEM, its link to transparency was also significant and positive (β = .17**), where it had been trending at best in the multiple regression (*p* = .11). Similar to the multiple regression results, trust recovery was not significantly related to monitoring (β = -.10^ns^).

Second, the SEM identified some significant relationships between the dispositional predictors and the behaviours. The most consistent finding came from attachment anxiety which was related to lower transparency (β = -.22***), lower monitoring (β = -.22***), lower remorse (β = -.21***), lower reconnection (β = -.17**), and lower explanation (β = -.25***). Trust propensity and attachment avoidance also had negative effects on remorse (β = -.12*; β = -.19**, respectively) and explanation (β = -.16*; β = -.23***, respectively).

We finally explored indirect and total effects of the dispositional predictors. *Trust propensity* had a significant total effect on trust recovery (i.e., the 95% CI did not include zero), *b* = -.16, *SE* = .077, 95% CI [-.31, -.005], β = -.13, which was partially explained by lower explanation (indirect effect: *b* = -.04, *SE* = .018, 95% CI [-.07, -.000], β = -.03).

*Attachment anxiety* also had a significant total effect on trust recovery, *b* = -.02, *SE* = .011, 95% CI [-.05, -.001], β = -.14. It showed significant indirect effect through transparency (*b* = -.006, *SE* = .003, 95% CI [-.012, -.001], β = -.04); remorse (*b* = -.01, *SE* = .004, 95% CI [-.02, -.003], β = -.06); reconnection (*b* = -.01, *SE* = .004, 95% CI [-.02, -.001], β = -.05); and explanation (*b* = -.01, *SE* = .003, 95% CI [-.02, -.002], β = -.05). Although not significant, attachment anxiety also showed a trending indirect effect through monitoring that went in the opposite direction, i.e., ameliorating trust recovery. Indeed, in the SEM attachment anxiety decreased monitoring and the residual effect of monitoring was trending into the direction of *decreasing* trust recovery (β = -.10, *p* = .10). The total effect of attachment anxiety could therefore be understood as a mostly negative influence on trust through decreased transparency, remorse, reconnection, and explanation, but also a – much smaller – positive influence through decreased monitoring.

**Table S1**

*Results of Structural Equation Modelling Testing the Relationship Between Dispositional Predictors (Attachment Anxiety, Attachment Avoidance, Trust Propensity) and Trust Recovery Through the Five Repair Behaviours*

| Regression | Estimate (b) | SE | z-test | 95% CI | Standard. β |
| --- | --- | --- | --- | --- | --- |
| Trust ~ |  |  |  |  |  |
| **Transparency** | **0.150**** | **0.054** | **2.792** | **[0.045, 0.255]** | **0.166** |
| Monitoring | -0.076 | 0.046 | -1.672 | [-0.165, 0.013] | -0.099 |
| **Remorse** | **0.261***** | **0.056** | **4.641** | **[0.151, 0.371]** | **0.280** |
| **Reconnection** | **0.249***** | **0.053** | **4.738** | **[0.146, 0.352]** | **0.275** |
| **Explanation** | **0.181**** | **0.057** | **3.158** | **[0.069, 0.293]** | **0.195** |
| Trust propensity | -0.054 | 0.071 | -0.763 | [-0.192, 0.084] | -0.045 |
| Anxiety | 0.006 | 0.011 | 0.533 | [-0.016, 0.028] | 0.034 |
| Avoidance | 0.004 | 0.013 | 0.284 | [-0.022, 0.030] | 0.017 |
| Transparency ~ |  |  |  |  |  |
| Trust propensity | -0.131 | 0.084 | -1.552 | [-0.296, 0.034] | -0.099 |
| **Anxiety** | **-0.042***** | **0.012** | **-3.429** | **[-0.066, -0.018]** | **-0.219** |
| Avoidance | -0.029 | 0.015 | -1.902 | [-0.059, 0.001] | -0.121 |
| Monitoring ~ |  |  |  |  |  |
| Trust propensity | -0.131 | 0.099 | -1.327 | [-0.326, 0.063] | -0.084 |
| **Anxiety** | **-0.050***** | **0.014** | **-3.423** | **[-0.078, -0.021]** | **-0.219** |
| Avoidance | -0.033 | 0.018 | -1.842 | [-0.068, 0.002] | -0.118 |
| Remorse ~ |  |  |  |  |  |
| **Trust propensity** | **-0.159*** | **0.080** | **-1.981** | **[-0.316, -0.002]** | **-0.124** |
| **Anxiety** | **-0.040***** | **0.012** | **-3.403** | **[-0.063, -0.017]** | **-0.214** |
| **Avoidance** | **-0.044**** | **0.015** | **-2.999** | **[-0.072, -0.015]** | **-0.189** |
| Reconnection ~ |  |  |  |  |  |
| Trust propensity | -0.061 | 0.086 | -0.710 | [-0.229, 0.107] | -0.046 |
| **Anxiety** | **-0.033**** | **0.013** | **-2.599** | **[-0.057, -0.008]** | **-0.170** |
| Avoidance | -0.012 | 0.016 | -0.786 | [-0.043, 0.018] | -0.051 |
| Explanation ~ |  |  |  |  |  |
| **Trust propensity** | **-0.200*** | **0.079** | **-2.539** | **[-0.354, -0.046]** | **-0.155** |
| **Anxiety** | **-0.046***** | **0.012** | **-3.999** | **[-0.069, -0.023]** | **-0.246** |
| **Avoidance** | **-0.053***** | **0.014** | **-3.708** | **[-0.081, -0.025]** | **-0.227** |
| Covariances |  |  |  |  |  |
| Trust prop. ~~ Anxiety | 0.388 | 0.000 |  | [0.388, 0.388] | 0.061 |
| Trust prop. ~~ Avoidance | -0.106 | 0.000 |  | [-0.106, -0.106] | -0.021 |
| Avoidance ~~ Anxiety | 4.480 | 0.000 |  | [4.480, 4.480] | 0.127 |

| Indirect and total effects | Estimate (b) | SE | 95% CI | Standard. β |
| --- | --- | --- | --- | --- |
| *Indirect effects of trust propensity through…* |  |  |  |  |
| Transparency | -0.020 | 0.014 | [-0.048, 0.009] | -0.016 |
| Monitoring | 0.010 | 0.010 | [-0.009, 0.029] | 0.008 |
| Remorse | -0.042 | 0.023 | [-0.086, 0.003] | -0.035 |
| Reconnection | -0.015 | 0.022 | [-0.058, 0.027] | -0.013 |
| **Explanation** | **-0.036** | **0.018** | **[-0.072, -0.000]** | **-0.030** |
| **Total effect of trust propensity** | **-0.156** | **0.077** | **[-0.308, -0.005]** | **-0.130** |
| *Indirect effects of attachment anxiety through…* |  |  |  |  |
| **Transparency** | **-0.006** | **0.003** | **[-0.012, -0.001]** | **-0.036** |
| Monitoring | 0.004 | 0.003 | [-0.001, 0.009] | 0.022 |
| **Remorse** | **-0.010** | **0.004** | **[-0.018, -0.003]** | **-0.060** |
| **Reconnection** | **-0.008** | **0.004** | **[-0.015, -0.001]** | **-0.047** |
| **Explanation** | **-0.008** | **0.003** | **[-0.015, -0.002]** | **-0.048** |
| **Total effect of anxiety** | **-0.023** | **0.011** | **[-0.046, -0.001]** | **-0.135** |
| *Indirect effects of attachment avoidance through…* |  |  |  |  |
| Transparency | -0.004 | 0.003 | [-0.010, 0.001] | -0.020 |
| Monitoring | 0.003 | 0.002 | [-0.001, 0.007] | 0.012 |
| **Remorse** | **-0.011** | **0.005** | **[-0.020, -0.003]** | **-0.053** |
| Reconnection | -0.003 | 0.004 | [-0.011, 0.005] | -0.014 |
| **Explanation** | **-0.010** | **0.004** | **[-0.017, -0.002]** | **-0.044** |
| Total effect of avoidance | -0.022 | 0.014 | [-0.050, 0.005] | -0.102 |

*Note*. Significant findings are indicated in bold. Confidence intervals are percentile bootstrap confidence intervals.

Finally, since the total effect of attachment avoidance on trust recovery was not significant, we did not interpret the indirect effects, as those would rely on different parts of (unshared) variance.

**References**

Rosseel, Y. (2012). Lavaan: An R package for Structural Equation Modeling. *Journal of Statistical Software*, *48*(2), 1-36. <https://doi.org/http://www.jstatsoft.org/v48/i02/>

Yzerbyt, V., Muller, D., Batailler, C., & Judd, C. M. (2018). New recommendations for testing indirect effects in mediational models: The need to report and test component paths. *Journal of Personality and Social Psychology*, *115*(6), 929-943. <https://doi.org/10.1037/pspa0000132>
